# Findings of ocular examinations in healthy full-term newborns

**DOI:** 10.5935/0004-2749.2021-0536

**Published:** 2022-07-04

**Authors:** Eşay Kıran Yenice, İkbal Seza Petriçli, Caner Kara

**Affiliations:** 1. Etlik Zübeyde Hanım Maternity and Women’s Health Teaching and Research Hospital, University of Health Sciences, Ankara, Turkey; 2. Dr Rıdvan Ege Hospital, Faculty of Medicine, Ufuk University Ankara, Turkey

**Keywords:** Eye abnormalities/diagnosis, Retinal, hemorrhage, Neonatal screening, Vision screening, Humans, Infant, newborn, Anormalidades do olho/diagnóstico, Hemorragia retiniana, Triagem neonatal, Seleção visual, Humanos, Re­cém-nascido

## Abstract

**Purpose:**

To assess the anterior and posterior segments of full-term neonates over a 1.5-year period.

**Methods:**

The findings of full-term neonates who underwent ophthalmological examinations between Jun^e 2019^ and December 2020 were analyzed, and the results were retrospectively recorded.

**Results:**

The study comprised 2972 neonates with a mean birth week of 38.7 ± 1.2 weeks and a mean birth weight of 3235 ± 464 g. The neonates were examined on an average of 49.3 ± 18.9 postnatal days. Of the examined neonates, 185 (6.2%) showed abnormal ophthalmological findings, the most prevalent of which were retinal hemorrhage in 2.3% (n=68) and white changes in the peripheral retina in 1.9% (n=55) of the neonates. Cases of optic disc pathologies (n=20), choroidal nevus (n=10), iris-choroidal coloboma (n=5), subconjunctival hemorrhage (n=6), non-specific retinal pigmentary change (n=4), congenital cataract (n=3), posterior synechia (n=3), iris nevus (n=3), corneal opacity (n=1), choroidal coloboma (n=1), iris coloboma (n=1), buphthalmos (n=1), anophthalmos (n=1), microphthalmia (n=1), lid hemangioma (n=1), and vitreous hemorrhage (n=1) collectively accounted for approximately 2% of all neonates. Pathologies that could potentially impair vision, which were detected by ophthalmological examination, accounted for 1.2% of all neonates (n=37).

**Conclusion:**

The most prevalent finding of the ophthalmological examinations of neonates in the present study was retinal hemorrhage. Ophthalmological examinations of neonates can help in identifying diseases that may affect their vision and are curable or may lead to amblyopia in the long term.

## Abstract (pt)

**Objetivo:**

Avaliar os segmentos anterior e posterior em recém-nascidos a termo durante um período de 1,5 anos.

**Métodos:**

Foram analisados recém-nascidos a termo que tiveram os olhos examinados entre junho d^e 2019^ e dezembro de 2020, e os resultados foram registrados retrospectivamente.

**Resultados:**

O estudo foi composto por 2.972 recém-nascidos com média de uma semana de nascimento de 38,7 ± 1,2 semanas e um peso médio ao nascer de 3235 ± 464 g. Os recém-nascidos foram examinados em média pós-natal de 49,3 ± 18,9 dias. Dos recém-nascidos, 185 (6,2%) apresentaram resultados oculares anormais. Os achados oculares anormais mais prevalentes foram hemorragia da retina em 2,3% (n=68) e alterações brancas na retina periférica em 1,9% (n=55) dos recém-nascidos. Casos de patologias de disco óptico (n=20), nevo de coroide (n=10), coloboma iris-coroide (n=5), hemorragia subconjuntival (n=6), alteração pigmentar da retina não específica (n=4), catarata congênita (n=3), Sinequia posterior (n=3), nevo da íris (n=3), opacidade da córnea (n=1), coloboma de coroide (n=1), coloboma de íris (n=1), buftalmos (n=1), anoftalmia (n=1), microftalmia (n=1), hemangioma de pálpebra (n=1) e hemorragia vítrea (n=1) contabilizaram coletivamente cerca de 2% dos recém-nascidos. As patologias que potencialmente prejudicam a visão, detectadas por exame ocular, representaram 1,2% dos recém-nascidos (n=37).

**Conclusão:**

O achado mais prevalente de exames oculares de recém-nascidos neste estudo foi hemorragia da retina. Exames oftalmológicos em recém-nascidos podem ser úteis na identificação de doenças que podem impactar a visão deles, podendo ser curáveis ou levar à ambliopia no longo prazo.

## INTRODUCTION

Neonates cannot express their distress vocally; thus, visual impairment in neonates must be diagnosed passively. The American Academy of Pediatrics supports conduction of red reflex examination as a part of ophthalmological evaluation of children in routine periodic visits, specifying that the examination should ideally begin during the neonatal period and continue throughout the routine periodic visits^(1)^. If any abnormalities are detected during these visits, the child should be referred to an ophthalmologist.

In Turkey, the Ministry of Health implemented the National Vision Screening Program and recommended visual screening for every neonate/child aged 0-3 months, 36-48 months, or enrolled in the first grade of primary education^(2)^. However, some pediatricians and family doctors are hesitant about the use of an ophthalmoscope to perform red reflex examinations in neonates. As a result, ophthalmic diseases are not diagnosed on time in some full-term neonates. Delayed diagnosis and the subsequent late initiation of the treatment of ophthalmic diseases in neonates can cause vision-threatening consequences. Thus, ophthalmological examination during the neonatal period may facilitate the early diagnosis and timely treatment of ophthalmic diseases^(3)^.

The present study aimed to identify the anterior and posterior segment findings that may impair ophthalmic health and vision in full-term neonates as well as highlight the prevalence of ophthalmic abnormalities in full-term neonates that were investigated during a 1.5-year period at our research center in Turkey.

## METHODS

The Ethical Review Committee of Etlik Zübeyde Hanım Maternity and Women’s Health Teaching and Research Hospital reviewed and approved this retrospective study, which was conducted in accordance with the standards of the Declaration of Helsinki for research involving human subjects. All study participants provided their informed consent. The necessary explanations regarding the study were provided in the parent’s preferred language using translators whenever necessary while obtaining informed consent. The families of the neonates were provided with an option of having the neonates undergo these examinations. The study included full-term neonates who underwent neonatal ophthalmological examination at the hospital between Jun^e 2019^ and December 2020. All neonates between 37 and 42 weeks of postmenstrual age were included in the study. Neonates born before 37 weeks of gestational age and those with severe systemic or syndromic disease were excluded from the study.

The examination was performed with the assistance of an ophthalmologist and a nurse. The neonates’ gestational age at birth and the birth weight was recorded, and information on the mothers’ pregnancy and delivery was also collected. At 1 h before the examination, the food intake was discontinued. The neonates’ pupils were then dilated using compound ophthalmic drops (0.5% tropicamide and 0.5% phenylephrine), which were administered every 5 min, 2 or 3 times until the pupils were dilated, beginning from 1 h before the examination. The anterior and posterior pictures of each eye were acquired using an indirect video ophthalmoscope and a 20-diopter lens. Next, a topical anesthetic was instilled, and a sterile pediatric eyelid speculum was placed in the eye for examination. The fundus was photographed in the following order: posterior pole, which included the optic nerve and macula, and the peripheral retinal fields. During or after any of the examination sessions, no ocular or systemic complications occurred. If an abnormality was detected in the anterior segment during indirect ophthalmoscopy, a comprehensive review was conducted using a hand-held slit-lamp biomicroscope (Shin-Nippon, Japan).

A follow-up examination was scheduled for neonates, whose ophthalmological examination revealed abnormal findings.

For statistical analysis, the SPSS 25.0 program package was applied. Data were presented as frequencies and percentages or as mean ± SD.

## RESULTS

A total of 2972 full-term neonates underwent neonatal ophthalmological examinations retrospectively, of which 1561 (52.5%) were males and 1411 (47.5%) were females. The neonates’ birth weight was 3235 ± 464 g, and their gestational age was 38.7 ± 1.2 weeks.

The neonates were examined at a mean 49.3 ± 18.9 (10-130) postnatal days. A total of 84.2% of the neonates were referred by a pediatrician, 12.6% by a family doctor, and 3.2% were brought to the examination at their families’ own will. Moreover, 1529 (51.45%) neonates were delivered vaginally and 1443 (48.55%) were delivered via Caesarian section.

**Figure 1:**
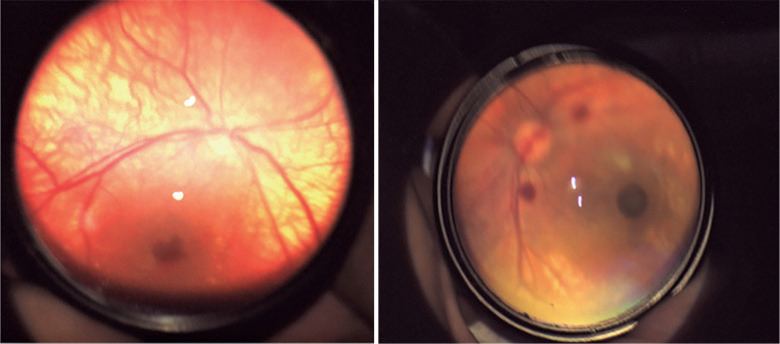
An illustrative case of retinal hemorrhage noted in 2.3% of all examined neonates.

Of the 2972 neonates who underwent ophthalmological examination, 185 (6.2%) presented with abnormal findings. Retinal hemorrhages were the most common finding among these, observed in 2.3% (n=68) of all screened neonates, and they were bilateral in 55.8% (n=8) of the neonates. Moreover, 61.7% (n=42) of all hemorrhages included the midperipheric and peripheric retina, whereas 38.2% (n=26) included the macula and peripheral retina together. At the 3-month follow-up, all retinal hemorrhages had resolved spontaneously. Retinal hemorrhages were also more frequent in the vaginal deliveries (n=60) in the present study. Figure 1 depicts retinal hemorrhages detected in neonates during the ophthalmological examination.

**Figure 2:**
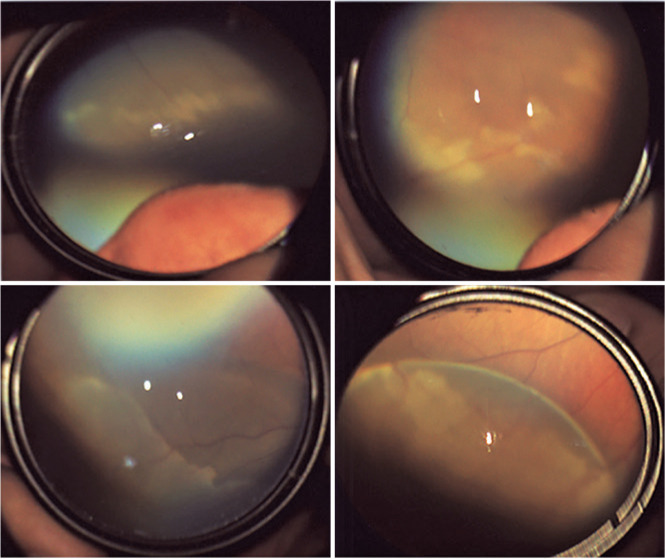
Retinal white spots recorded in 1.9% of all screened neonates.

The second-most common finding was white changes in the retina, which was recorded in 1.9% (n=55) of all screened neonates (Figure 2). Cases of retinal white changes in various forms, such as a spot, strip, or patch, were also recorded.

**Figure 3:**
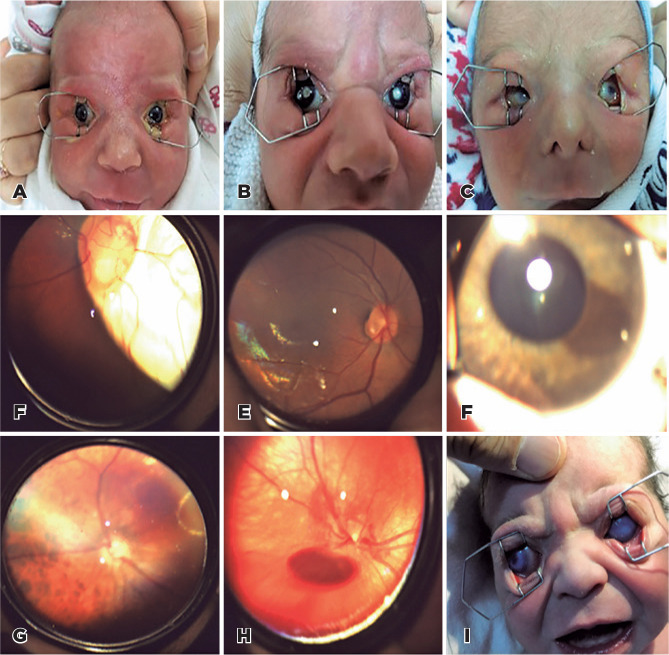
Other ocular abnormalities detected in 1.9% of all screened neonates. (A) Iris coloboma. (B) Congenital cataract. (C) Congenital microphthalmia. (D) Optic disc coloboma. (E) Optic pit. (F) Iris nevus. (G) Choroidal nevus. (H) Vitreous Hemorrhage. (I) Buphthalmos.

As listed in Table 1, the other abnormalities included subconjunctival hemorrhage, isolated bilateral iris coloboma (Figure 3A), congenital cataracts (Figure 3B), microphthalmia (Figure 3C), optic disc-choroidal coloboma (Figure 3D), optic disc pathologies (Figure 3E), iris nevus (Figure 3F), choroidal nevus (Figure 3G), vitreous hemorrhage (Figure 3H), buphthalmos with an intraocular pressure of 40 mmHg (Figure 3I), non-specific retinal pigmentary changes, posterior synechia, corneal opacity, and anophthalmus.

The rate of serious pathologies diagnosed by ophthalmological examination that could affect vision, such as cataract (0.1%), macular hemorrhage (0.9%), optic disc-choroidal coloboma (0.03%), vitreous hemorrhage (0.03%), and morning glory (0.03%) were among the disorders detected on ophthalmological examination (1.2%). Two of three neonates with congenital cataract were affected bilaterally, and one was affected unilaterally. Two of these neonates were referred by a pediatrician, whereas one of the neonates with bilateral congenital cataracts came for the evaluation at the families’ own will, and all neonates underwent the prescribed surgery.

Based on the results, corneal opacity is attributable to idiopathic causes after excluding other causes, such as infections and metabolic, genetic, and developmental factors.

Follow-up observation revealed vitreous hemorrhages to have spontaneously resolved without requiring any intervention.

## DISCUSSION

The 1.5-year outcomes of the investigation of 2972 healthy full-term neonates have been presented in this study in an attempt to identify neonates with ophthalmic disorders at an early stage. In several countries, neonatal ophthalmological screening is not widely performed. However, in Turkey, the Ministry of Health implemented the National Vision Screening Program, which provides information regarding ophthalmological screening from a young age as well as the age ranges that mandates screening.

The American Academy of Pediatrics, the American Association for Pediatric Ophthalmology and Strabismus, and the American Academy of Ophthalmology advocate red reflex evaluation in neonates, infants, and children^(4,5)^. Red reflex testing is a straightforward technique; however, some clinicians are hesitant to use an ophthalmoscope to perform red reflex examinations in neonates^(6)^; therefore, they refer neonates to a pediatric ophthalmology clinic for detailed examinations, which simply increases the number of patients to be evaluated. Neonatal ophthalmological examination, including indirect binocular ophthalmoscopy, is performed after pupillary dilation for all neonates referred to our center for medicolegal issues.

Compared with other disorders that were screened during the neonatal period, congenital ophthalmic diseases (1:2700)^(7)^ are reportedly more prevalent^(8-11)^. In lieu of the benefits and risks associated with perinatal ophthalmological examinations, we believe that vision screening programs can play a positive role in detecting congenital ophthalmic diseases in healthy neonates.

The clinical importance of ocular abnormalities in the examined neonates varied. For instance, subconjunctival hemorrhage, retinal pigmentation, self-resolved retinal hemorrhage, vision-threatening macular hemorrhage, large physiological cupping of the optic disc, congenital disc anomalies, choroidal nevus and coloboma, optic pit, severe cataract, and vitreous hemorrhage are some of the diseases listed in order of their clinical importance. Importantly, clinically significant anomalies should be monitored periodically because they can affect vision with a long-term risk of amblyopia or may necessitate some type of intervention^(12)^.

Retinal hemorrhages may interfere with the appropriate development of vision and may thus impair the functioning of the visual pathway^(13,14)^. Previous researches have reported that the most prevalent ocular abnormality is retinal hemorrhage, with prevalences of 21.5%, 2.4%, and 20.3%, respectively^(15-17)^. In accordance with literature, the most common examination finding of neonates in our study was retinal hemorrhage, with a prevalence of 2.3%.

Additionally, the chances of examining neonates in the first month of birth are very low, as the neonates were referred for examination at nearly the third month of birth. Therefore, considering that retinal hemorrhages are transient, delays in examination may affect and inaccurately reduce the prevalence of retinal hemorrhages. The prevalence of retinal hemorrhage increases with early examinations. Because hemorrhages mostly occurred in the mid-peripheral and peripheral areas (61.7%) when compared to that in the macular area (38.2%) and the mean postnatal examination duration was late in the present study, all retinal hemorrhages were resolved spontaneously without intervention 3 months after the follow-up. Therefore, retinal hemorrhages were less likely to affect vision development in the neonates in our study group. Long-term follow-up is however warranted to clarify this situation.

The second-most common result was the appearance of peripheral retinal white patches or areas, which accounted for 1.9% of all screened neonates in this study. The peripheral retina exhibited white spots, strips, or patches. Ma et al. hypothesized that this type of retinal alteration may be caused by a variety of factors, including the following: 1) decreased blood supply to the peripheral retina throughout development that results in retinal malnutrition-like alterations; 2) a delay in the formation of retinal vascular epithelial cells in the periphery compared with that in the posterior areas, which results in retinal exudations owing to the immature blood-retina barrier; and 3) subretinal lipid or calcium deposition, which partially resolves retinal hemorrhage, and local underdeveloped retinal pigmentary epithelium^(12)^. In this study, we found that most cases of retinal white changes did not pose any concern and can hence be classified as a physiological modification.

Congenital cataract is one of the most common causes of treatable blindness. Early intervention is therefore recommended to avoid the occurrence of amblyopia and nystagmus due to visual deprivation. The recommended period for conducting surgery to prevent amblyopia is during the first 4 weeks of life for unilateral cataract and in the first 6 weeks of life for bilateral cataract^(18,19)^. In our study, one neonate showed bilateral congenital cataract and was accordingly recommended surgery as an early intervention.

Neuroimaging, endocrine investigations, or detailed genetic testing are recommended for the early diagnosis of untreatable disorders, such as congenital anomalies of the optic disc and microphthalmia^(20,21)^. In our study, the neonates were referred for further investigation. Early ophthalmological screening may therefore help improve neonatal well-being by prompting investigations of the systemic association, treatment designing, and possible rehabilitation of diseases.

The study’s limitation was that it focused on cases within a single center. As a result, a multicenter re­search with a large sample size and long-term follow-up is warranted to examine the incidence and etiology of ocular anomalies.

Thus, neonatal ophthalmological examinations can help identify pathologies that potentially impact vision and are curable or may lead to amblyopia in the long term.
